# Left ventricular metastasis from tongue squamous cell carcinoma presenting with ventricular tachycardia: a case report

**DOI:** 10.1093/ehjcr/ytag031

**Published:** 2026-03-06

**Authors:** So Hirata, Satoshi Higuchi, Saeko Yoshizawa, Morio Shoda, Junichi Yamaguchi

**Affiliations:** Department of Cardiology, Tokyo Women’s Medical University, 8-1 Kawadacho, Shinjuku-ku, Tokyo 162-8666, Japan; Department of Cardiology, Saiseikai Kumamoto Hospital, 5-3-1 Chikami, Minami-ku, Kumamoto 861-4193, Japan; Department of Cardiology, Tokyo Women’s Medical University, 8-1 Kawadacho, Shinjuku-ku, Tokyo 162-8666, Japan; Clinical Research Division for Heart Rhythm Management, Tokyo Women’s Medical University, 8-1 Kawadacho, Shinjuku-ku, Tokyo 162-8666, Japan; Department of Cardiology, Tokyo Women’s Medical University, 8-1 Kawadacho, Shinjuku-ku, Tokyo 162-8666, Japan; Department of Surgical Pathology, Tokyo Women’s Medical University, 8-1 Kawadacho, Shinjuku-ku, Tokyo 162-8666, Japan; Department of Cardiology, Tokyo Women’s Medical University, 8-1 Kawadacho, Shinjuku-ku, Tokyo 162-8666, Japan; Clinical Research Division for Heart Rhythm Management, Tokyo Women’s Medical University, 8-1 Kawadacho, Shinjuku-ku, Tokyo 162-8666, Japan; Department of Cardiology, Tokyo Women’s Medical University, 8-1 Kawadacho, Shinjuku-ku, Tokyo 162-8666, Japan

**Keywords:** Onco-cardiology, Metastatic cardiac cancer, Ventricular arrhythmia, Tongue squamous cell carcinoma, Left ventricle, Non-sustained ventricular tachycardia, Case report

## Abstract

**Background:**

Cardiac metastasis from tongue squamous cell carcinoma (TSCC) is extremely rare. Endocardial involvement, particularly in the left heart, occurs in only 3%–6% of cases and is often asymptomatic until advanced stages. Early detection is challenging but critical to prevent life-threatening complications.

**Case summary:**

A 75-year-old man with a history of TSCC presented with non-sustained ventricular tachycardia (NSVT) detected during routine post-operative surveillance. Transthoracic and transoesophageal echocardiography, cardiac magnetic resonance imaging, and contrast-enhanced computed tomography revealed a well-defined mass in the left ventricular cavity infiltrating the anterolateral papillary muscle. Surgical excision with concomitant mitral valve replacement was performed. Histopathology confirmed metastatic TSCC. Post-operatively, ventricular arrhythmias resolved completely, and the patient remained recurrence-free with preserved cardiac function during follow-up.

**Discussion:**

This case highlights the rare presentation of left-sided endocardial cardiac metastasis from TSCC manifesting as potentially life-threatening ventricular arrhythmias. It emphasizes the importance of considering cardiac metastasis in cancer patients with new-onset arrhythmias, demonstrates the diagnostic utility of multi-modal imaging, and underscores the therapeutic benefit of timely surgical intervention in selected patients.

Learning points

- Cardiac metastasis should be considered a potential cause of ventricular arrhythmias in patients with tongue squamous cell carcinoma.
- In cancer patients with new-onset ventricular arrhythmias, multi-modal imaging may facilitate early diagnosis and guide potentially life-saving interventions.

## Introduction

Cardiac tumours are rare, but metastatic involvement is far more common than primary cardiac tumours. Autopsy studies report cardiac metastases in 1.5%–20% of patients with malignancy,^1,2^ most frequently from lung, breast, melanoma, lymphoma, or leukaemia.^3,4^ Metastases can occur via direct invasion, haematogenous, lymphatic, or transvenous spread, and early diagnosis is challenging due to non-specific symptoms. Cardiac involvement from tongue squamous cell carcinoma (TSCC) is extremely rare, with only isolated reports.^5^ Tongue squamous cell carcinoma typically metastasizes to cervical lymph nodes, lungs, or bone; cardiac metastases—especially involving the left-sided endocardium or myocardium—are highly atypical.^6^ When clinically evident, cardiac metastases may present with arrhythmias or embolic events. Multi-modality imaging, including magnetic resonance imaging (MRI) and positron emission tomography–computed tomography (PET–CT), is essential for delineating tumour extent and differentiating from thrombi.^7,8^ Although management is usually palliative, surgical intervention may be considered in select cases to relieve symptoms or address life-threatening complications such as arrhythmias.

## Summary figure

**Figure:**
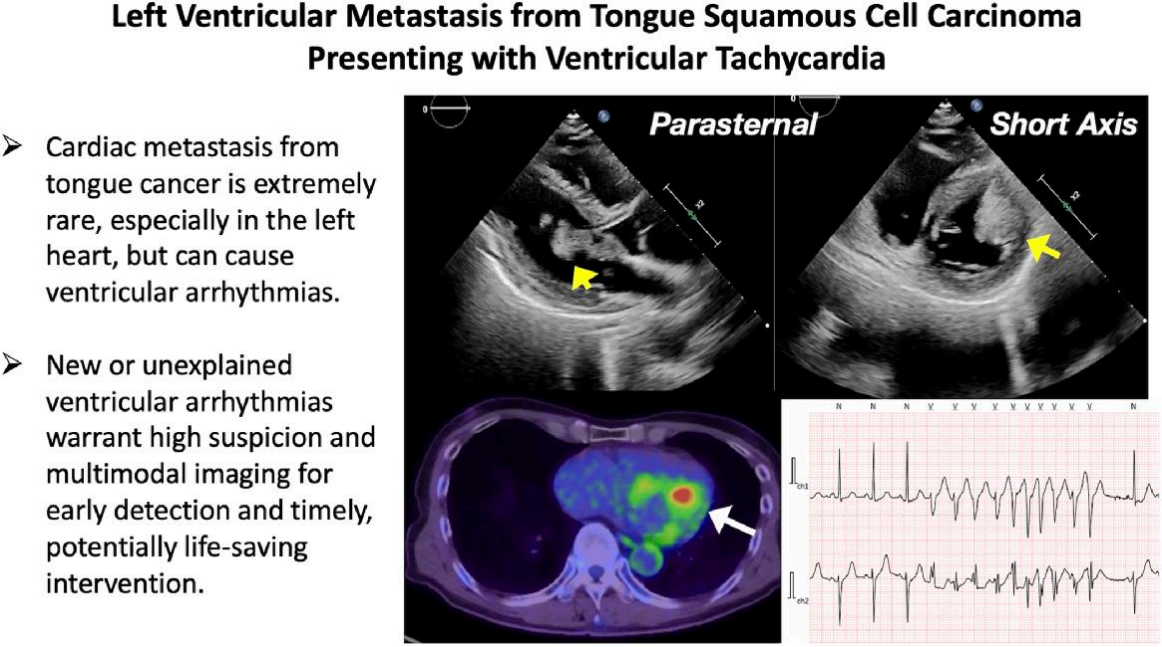


## Case presentation

A 75-year-old man with a history of TSCC presented with palpitations during routine post-operative surveillance. One year earlier, he had undergone partial glossectomy and cervical lymphadenectomy for primary TSCC, followed by bilateral pulmonary metastasectomy 3 months prior. He had no history of hypertension, diabetes mellitus, dyslipidaemia, or ischaemic heart disease.

**Figure 1:**
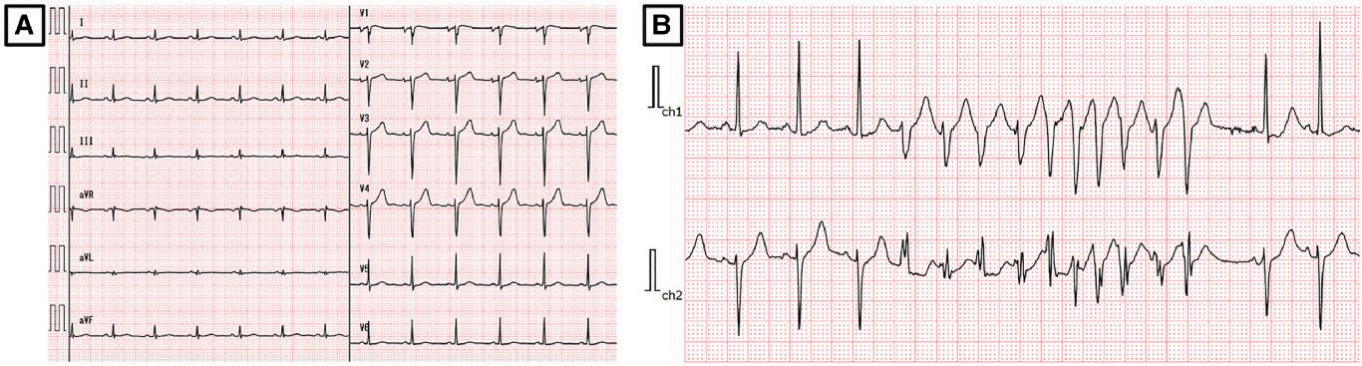
Electrocardiographic findings. (*A*) A 12-lead electrocardiogram showed sinus rhythm at 78 beats per minute, with normal axis, no significant ST-T changes, and normal R-wave progression. (*B*) Ambulatory Holter monitoring revealed frequent premature ventricular contractions and multiple episodes of non-sustained ventricular tachycardia.

**Figure 2:**
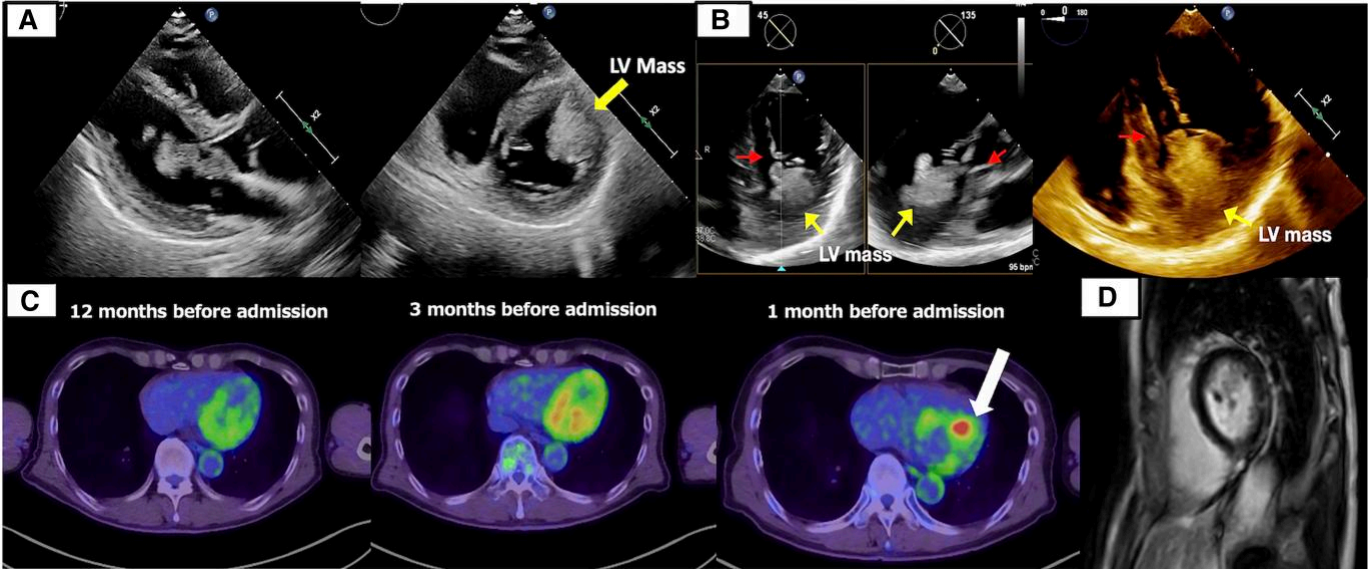
Multi-modal imaging of the left ventricular mass. (*A*) Transthoracic echocardiography revealed an irregularly shaped mass within the left ventricle. (*B*) Transoesophageal echocardiography demonstrated two cord-like structures measuring 20 and 19 mm (red arrows) extending from the mass. Part of the mass appeared to involve the left ventricular papillary muscles (yellow arrow). (*C*) Serial positron emission tomography–computed tomography images demonstrated increased metabolic activity in the left ventricle (SUVmax 7.12, white arrow) on the scan obtained 1 month prior to admission, compared with an earlier study, consistent with progressive tumour involvement. (*D*) Cardiac magnetic resonance imaging demonstrated no late gadolinium enhancement.

**Figure 3:**
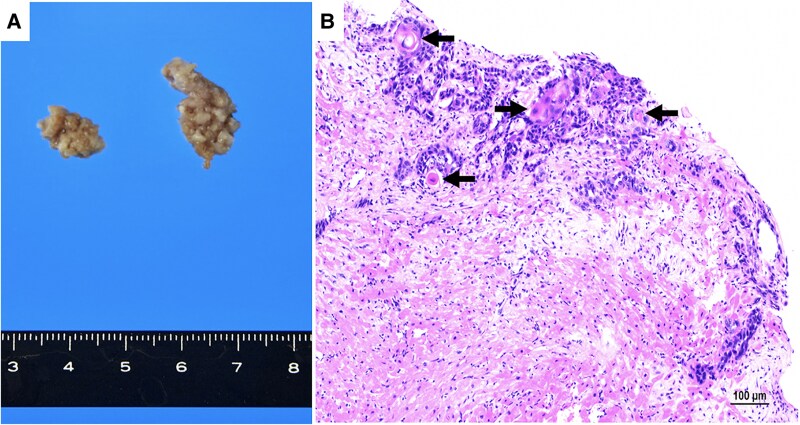
Intra-operative and histopathological findings. (*A*) Gross specimen of the tumour mass. (*B*) Histopathological examination showing keratin pearls (green arrow) and cells with a high nuclear-to-cytoplasmic ratio, consistent with squamous cell carcinoma.

During follow-up, continuous electrocardiographic monitoring revealed frequent premature ventricular contractions (PVCs) and multiple episodes of non-sustained ventricular tachycardia (NSVT), prompting referral to cardiology. On presentation, vital signs were within normal limits, and physical examination was unremarkable, with no murmurs, gallops, or signs of volume overload. A 12-lead electrocardiogram showed sinus rhythm at 78 b.p.m. without significant ST-T changes (*Figure 1A*). Ambulatory Holter monitoring recorded 2146 PVCs over 24 h and 115 episodes of NSVT, with a peak heart rate of 208 b.p.m. during runs of up to 16 consecutive beats (*Figure 1B*). Laboratory findings were unremarkable except for an elevated squamous cell carcinoma (SCC) level of 4.1 ng/mL (reference range: <1.5 ng/mL). Chest X-ray demonstrated clear lung fields and no cardiomegaly. Transthoracic echocardiography revealed a 3.3 × 2.3 cm irregular mass in the left ventricle, appearing attached to and infiltrating the anterolateral papillary muscle, with a mildly reduced left ventricular ejection fraction (LVEF) of 48% (normal >50%) (*Figure 2A*; see Supplementary material online, *Video S1*). Transoesophageal echocardiography (TEE) provided detailed visualization of the mass and identified two cord-like structures measuring 20 and 19 mm, consistent with tumour infiltration or thrombotic appendages (*Figure 2B*; see Supplementary material online, *Video S2*). Retrospective review of serial PET–CT scans obtained 12, 3, and 1 month prior to admission revealed a progressively increasing 18F-fluorodeoxyglucose (FDG) uptake in the left ventricular mass, preceding the onset of ventricular arrhythmias (*Figure 2C*). Cardiac magnetic resonance imaging (CMR) was performed to assess tissue characterization and arrhythmogenic potential; late gadolinium enhancement (LGE) was absent (*Figure 2D*). Furthermore, coronary angiography demonstrated no significant stenosis, excluding an ischaemic contribution as the cause of NSVT (see Supplementary material online, *Figure S1*). Given the high risk of malignant arrhythmias and potential sudden cardiac death, a multi-disciplinary team of cardiologists, cardiac surgeons, and oncologists formulated a surgical plan. The patient underwent resection of the intra-cardiac mass with concomitant mitral valve replacement. Gross specimen of the tumour mass was obtained (*Figure 3A*). Intra-operative findings confirmed extensive infiltration of the anterolateral papillary muscle, necessitating careful reconstruction of the sub-valvular apparatus. Histopathological examination confirmed metastatic SCC, characterized by sheets of tumour cells exhibiting keratinization (*Figure 3B*).

Post-operative recovery was uneventful. Holter monitoring performed after surgery showed complete resolution of NSVT without the use of any antiarrhythmic medication. The patient remained haemodynamically stable and was discharged with preserved left ventricular function. Adjuvant systemic chemotherapy was planned to address potential residual disease and reduce the risk of further metastasis. Follow-up at 3 months demonstrated no recurrence of arrhythmia or cardiac mass on imaging.

## Discussion

Cardiac metastasis, although uncommon, represents a clinically significant complication in patients with advanced malignancies. This case highlights a particularly rare presentation: ventricular arrhythmias caused by myocardial involvement from TSCC. It underscores the diagnostic challenges and the critical role of multi-disciplinary collaboration in managing such complex presentations.

While TSCC commonly metastasizes to regional lymph nodes, lungs, and bones, cardiac involvement is rare. Autopsy studies report cardiac metastases in 10%–12% of cancer patients^2,3^; however, myocardial involvement from head and neck cancers is extremely infrequent. In a large autopsy series, <1% of cardiac metastases were attributed to head and neck tumours.^5^

In the present case, a left ventricular mass infiltrating the anterolateral papillary muscle provided a plausible substrate for the observed NSVT and frequent PVCs. Although the precise mechanism of VT initiation remains uncertain, tumour invasion may disrupt myocardial architecture, leading to conduction slowing and repolarization abnormalities that enhance arrhythmogenic potential. The tumour–myocardium interface can thus generate electrophysiological heterogeneity, predisposing to re-entry circuits that may result in sustained or non-sustained VT.^5,9,10^ These observations highlight the dual role of cardiac tumours as both structural distorters and active arrhythmogenic substrates. Although CMR did not demonstrate LGE—a typical marker of fibrosis or scarring—functional arrhythmogenic substrates cannot be excluded. Positron emission tomography–computed tomography confirmed high metabolic activity at the lesion, consistent with active tumour tissue.

Arrhythmias are a known manifestation of cardiac metastases and may range from atrial fibrillation to ventricular tachycardia and sudden cardiac death, depending on the anatomical location of involvement.^11^ Papillary muscle infiltration, as in this case, is particularly relevant due to its arrhythmogenicity and mechanical role in valve function.

Several previous reports have described similar presentations, including cases of complete atrioventricular block as the initial manifestation of cardiac metastasis from oral cavity cancer, sudden death due to conduction system invasion in tongue carcinoma, and syncope as the first symptom of early cardiac metastasis from TSCC.^12,13^ Collectively, these cases suggest that myocardial or septal infiltration by metastatic lesions can lead to conduction disturbances and various arrhythmias. Although such occurrences remain exceedingly rare, arrhythmia as an initial manifestation of metastatic cardiac tumour originating from tongue cancer is clinically significant and warrants recognition and reporting.

This case emphasizes the value of multi-modal imaging. This case emphasizes the value of multi-modal imaging. Transthoracic echocardiography and TEE identified the intra-cardiac mass and its structural effects. Positron emission tomography–computed tomography confirmed malignancy through metabolic activity, and CMR provided tissue characterization, helping to exclude infiltrative cardiomyopathies such as sarcoidosis or amyloidosis.^7,8^ Positron emission tomography–computed tomography has increasingly been recognized for distinguishing malignant from benign cardiac lesions, with high sensitivity and specificity, particularly in metabolically active tumours.^8^

Given the high risk of malignant arrhythmias and sudden cardiac death, surgical resection was considered both diagnostic and therapeutic. Mitral valve replacement was required due to tumour infiltration of the anterolateral papillary muscle. Post-operative resolution of ventricular arrhythmias supports a causal relationship between the tumour and electrical instability, consistent with prior reports where tumour reduction—surgically or systemically—led to rhythm stabilization.^14^

This case highlights the need for clinicians to remain attentive to new cardiovascular symptoms, such as palpitations or arrhythmias, in patients with known malignancies, even in the absence of prior cardiac disease. It also illustrates how a multi-disciplinary approach, incorporating advanced imaging and timely intervention, can clarify diagnosis, guide treatment, and provide symptom relief. While cardiac metastases remain rare, awareness of such presentations can inform clinical decision-making and enhance educational understanding of the interplay between malignancy and cardiac physiology.

## Conclusion

This case underscores that cardiac metastasis should be considered a potential cause of ventricular arrhythmias in patients with TSCC, even in the absence of prior cardiovascular disease. Early assessment with multi-modal cardiac imaging, including transthoracic echocardiography, is essential for timely diagnosis.

## Lead author biography


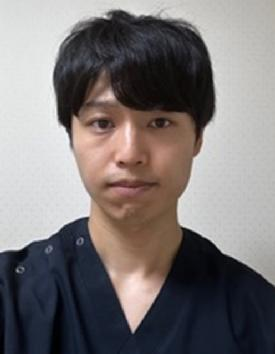


Dr So Hirata is a cardiology fellow in the Department of Cardiology at Saiseikai Kumamoto Hospital. His professional interests include general medicine, hospitalist care, and general cardiology. He is also an active member of the American College of Physicians, the European Society of Cardiology, and the American Heart Association.

## Supplementary Material

ytag031_Supplementary_Data

## Data Availability

The data underlying this article cannot be shared publicly due to the privacy of the individual who participated in the study. The data will be shared on reasonable request to the corresponding author.

## Supplementary material

**Consent:** The patient consented to the publication of this case report in compliance with the COPE guidelines.
