# Supplementary material for: Left ventricular metastasis from tongue squamous cell carcinoma presenting with ventricular tachycardia: a case report
Source: Eur Heart J Case Rep. 2026 Mar 6;10(5):ytag031. doi: 10.1093/ehjcr/ytag031 (PMC13158794; doi:10.1093/ehjcr/ytag031)
Supplement: ytag031_Supplementary_Data [file ytag031_supplementary_data.zip › Supplemental Material.docx]

**Supplemental Material**

**
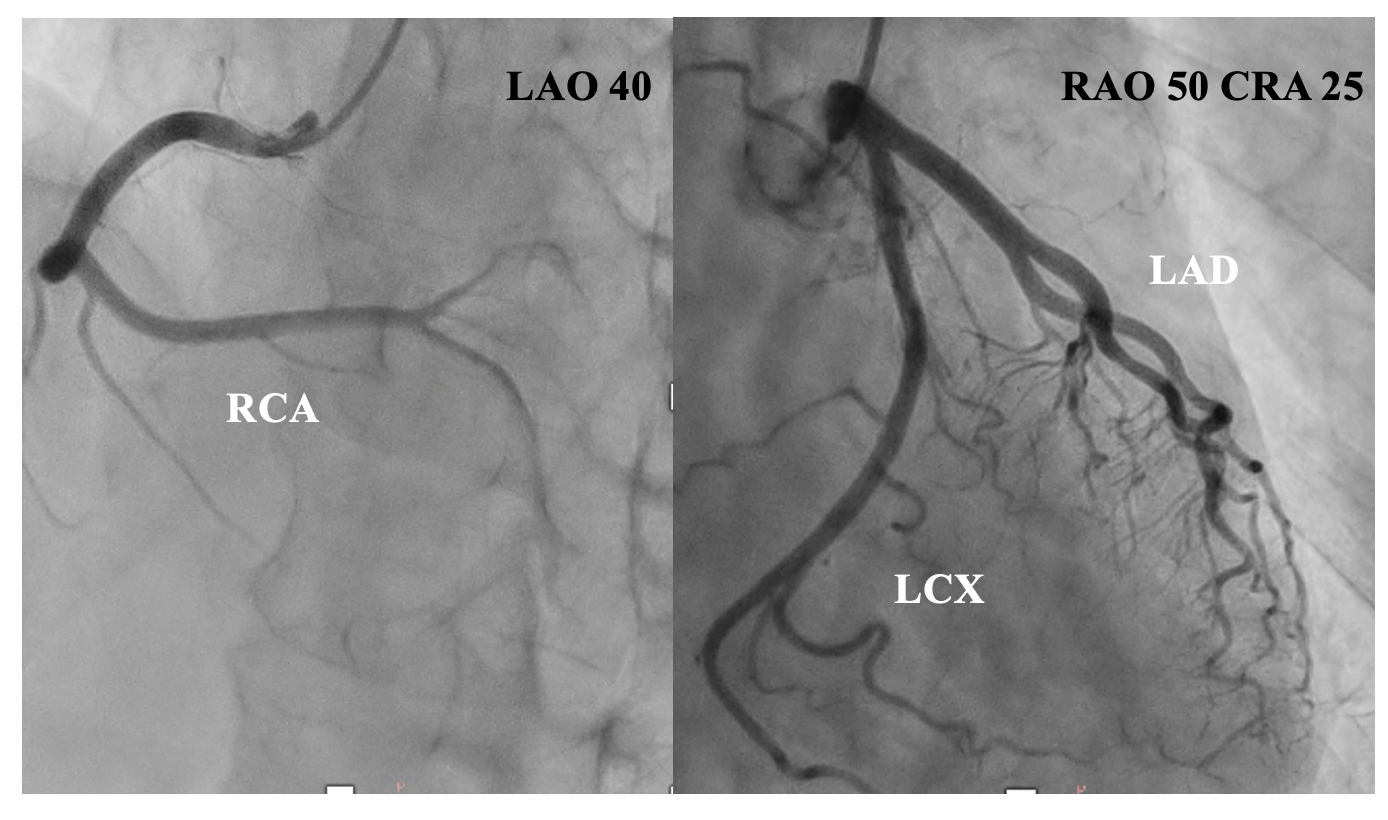
**

**Supplemental Figure 1. Coronary angiography.**
Coronary angiography revealed no significant stenosis in any of the major coronary arteries.
RCA = right coronary artery; LCX = left circumflex artery; LAD = left anterior descending artery; LAO = left anterior oblique; RAO = right anterior oblique.

**Supplemental Video 1. Transthoracic echocardiography.**
(A) Long-axis view. (B) Short-axis view.

**Supplemental Video 2. Transesophageal echocardiography.**
(A) 0° view. (B) 45° and 135° views.
